# Repeated Epley’s maneuver in the same session in benign positional paroxysmal vertigo

**DOI:** 10.1016/S1808-8694(15)30106-3

**Published:** 2015-10-19

**Authors:** Gustavo Polacow Korn, Ricardo S. Dorigueto, Maurício Malavasi Ganança, Heloísa Helena Caovilla

**Affiliations:** a M.S. in Otorhinolaryngology - UNIFESP/EPM (Paulista School of Medicine – Federal University of São Paulo).; b M.S. in Otorhinolaryngology - UNIFESP/EPM.; c Full Professor of Otorhinolaryngology - UNIFESP/EPM.; d Speech and Hearing Therapist, Associate Professor – Discipline of Neurotology – Department of Otorhinolaryngology-Head and Neck Surgery UNIFESP/EPM.

**Keywords:** labyrinth, physiologic nystagmus, semicircular canals, vertigo

## Abstract

**Aim:**

To assess whether more than one Epley’s maneuver in the same session, compared to a single one, decreases the number of sessions necessary to suppress positional nystagmus.

**Method:**

Epley’s maneuver was done in 123 patients with BPPV due to unilateral posterior semicircular canal canalolithiasis. The number of sessions for positional nystagmus suppression was compared in two groups of patients. Group I consisted of 75 patients submitted to a single Epley’s maneuver on weekly sessions and group II consisted of 48 patients that were submitted to four Epley’s maneuvers during the first session.

**Results:**

Group II showed greater nystagmus latency and duration than group I (p<0.05). The number of sessions and standard deviation showed by group I was greater than in group II (p=0.008). We observed a significant association between number of sessions and group (p=0.039) studied. Group II had 21.4% more nystagmus-free patients following only one session (CI95% [7.7% - 35.1%]).

**Conclusion:**

Repeated Epley’s maneuvers in less sessions rendered more positional nystagmus-free patients when compared to those submitted to more sessions of single maneuvers.

## INTRODUCTION

Benign paroxysmal positional vertigo (BPPV) is the most common cause of peripheral vertigo^1^, with an incidence that varies between 11 and 64 cases per 100 mil^2^, ^3^, predominantly in the age range between 50 and 55 years in idiopathic cases^4^; and very rarely in childhood^5^.

Symptoms were present for an average of 30 months before therapeutic intervention in patients with BPPV^6^.

BPPV is characterized by brief spells of vertigo, nausea and/or positional nystagmus at head position change^7^. After the vertigo spell, a vague feeling of floating-like dizziness may persist for hours, or even days; the intensity of BPPV clinical manifestations and its recurrent character may impact a patient’s professional, social, domestic and even school activities ^7^.

BPPV may be triggered by a head injury, infectious labyrinthitis, vertebro-basilar insufficiency, after ear surgery, endolymphatic hydrops, vestibular neuritis, or middle ear disease; however, in most of the cases it is idiopatic^1^, ^8^.

As to the physiopathology, there are two theories: cupulolithiasis, in which statocone debris are attached to the cupulla^9^, and canalolithiasis, in which the debris float freely in the endolymph along the semicircular canal involved^10^.

The theory of posterior canal canalolithiasis is considered the most convincing one, explaining BPPV’s pathogenesis and one that is supported by the efficiency of specific therapeutic maneuvers^11^.

BPPV most frequently affects the posterior semicircular canal^1^; however, it may also involve the anterior^7^ or the lateral^1^ canals. BPPV may involve the labyrinth bilaterally or affect different canals simultaneously^12^. In patients with BPPV affecting the posterior canal, the right side has been 1.41 times more frequently involved when compared to the left one, and the habit of sleeping on one’s right side may be a possible explanation^13^.

The canal involved may be identified by the very characteristics of the positional nystagmus. Nystagmus and vertigo usually happen after a latency of some seconds, has a limited duration and is fatigable by repeating the provocative maneurver^14^, and, in the absence of such characteristics, one should consider a vertigo of central cause^4^. The most often used diagnostic procedures are the Dix-Hallpike and Brandt-Daroff tests and the study of positional nystagmus towards the lateral canal^7^. The vertical, upwards and rotational nystagmus points towards an involvement of the posterior semi-circular canal^11^.

In BPPV, vertigo may happen dissociated from nystagmus. Nystagmus may be absent because of interferences of habituation processes, or because the triggering head movement is enough to cause vertigo, however does not reach the necessary stimulation threshold to trigger ocular movement^15^.

BPPV may resolve spontaneously in untreated patients^16^, ^17^, and this could be explained by the low calcium concentration (20mM) in endolymph, which would dissolve the statocone debris^18^, or by avoiding the triggering positions^19^. Untreated patients may improve with time, however, patients treated with the Epley’s maneuver had five times greater chance of symptom resolution and Dix-Hallpike negative test in their first return visit^19^.

BPPV may be treated with the use of therapeutic maneuvers, that would move the statocone debris back to the utricle^20^, ^21^. Epley’s maneuver (1992) is one the treatments for BPPV in the posterior semicircular canal^7^. Vertigo and positional nystagmus resolution happened in all BPPV cases; notwithstanding, 10.0% of the cases continued to present atypical symptoms, suggesting a concurrent problem, and 30.0% presented one or more recurrences, which responded well to the new treatment^21^. We considered the eventual need for more than one session of maneuver^22^, ^23^ and the possibility for recurrencies^16^, ^23^.

In the posterior semicircular canal BPPV, different therapeutic approaches used one single Epley’s maneuver per session^24^, ^25^, ^26^, ^27^.

The original proposal of particles repositioning for the treatment of BPPV in the posterior semicircular canal already advocated procedure repetition in the same session, from one to five times, until nystagmus was no longer seen; the procedure should be repeated weekly, until both vertigo and nystagmus stop^21^.

We proposed repetitive maneuvers in one single session, until nystagmus was no longer seen^28^, ^29^, ^30^, ^31^ or until vertigo and nystagmus stopped^23^, ^32^, ^33^, ^34^, without comparing Epley’s maneuver efficacy repeated in the same session with one single maneuver per session.

Patients with posterior canal canalolithiasis underwent repositioning maneuver repetition in one single session. When the Dix-Hallpike test became negative 20 minutes after the maneuver, the treatment was considered a success, and when it remained positive, a second maneuver was carried out after 20 minutes. Following, if the positional nystagmus persisted, up to four additional maneuvers were carried out in the same session, which were well tolerated by the patients. We did not find statistically significant differences in efficacy between one single session of repeated maneuvers and one session with one single maneuver. The elimination of vertigo and nystagmus with maneuvers repetition in the same session is clinically more convenient, because it allows to show the patient the treatment efficacy for there is a progressive symptoms improvement during the procedure.

Facing the scarcity of related studies in this area, there was this interest in verifying the clinical evolution of patients with BPPV submitted to the Epley’s maneuver repeated in the same session.

The goal of the present investigation is to assess repeated Epley’s maneuver in one same session and see if it results in less sessions necessary to completely abolish positional nystagmus when compared to one single Epley’s maneuver per session.

## METHOD

This prospective case-controled study, approved by the Ethics in Research Committee, includes 123 patients with vertigo and diagnostic hypothesis of BPPV.

We included patients with BPPV by unilateral posterior semicircular canal canalolithiasis who complained of vertigo and positional nystagmus of latency and duration of less than 1 minute and fatigable at the Dix-Hallpike test. Exclusion criteria in the study were:

1) anterior or lateral semicircular canal involvement;
2) nystagmus lasting for more than 1 minute, characterizing cupulolithiasis;
3) signs and symptoms of central nervous system involvement;
4) hearing involvement, unless if matching criteria for presbycusis;
5) bilateral involvement of posterior semicircular canal;
6) physical restrictions that would prevent the diagnostic or treatment maneuvers;
7) patients with dizziness only, without positional at the diagnostic maneuver;
8) use of medication that could influence the vestibular system.

For the positional nystagmus investigation we carried out the Dix-Hallpike test^36^.

The patient’s ocular movements were observed with the help of Frenzel’s goggles. The test was started at the position that triggered the vertigo and/or the nystagmus, according to information obtained from each patient. If the patient did not know how to report on which position would be responsible for vertigo onset, the maneuver started on the right side.

**Chart 1:**
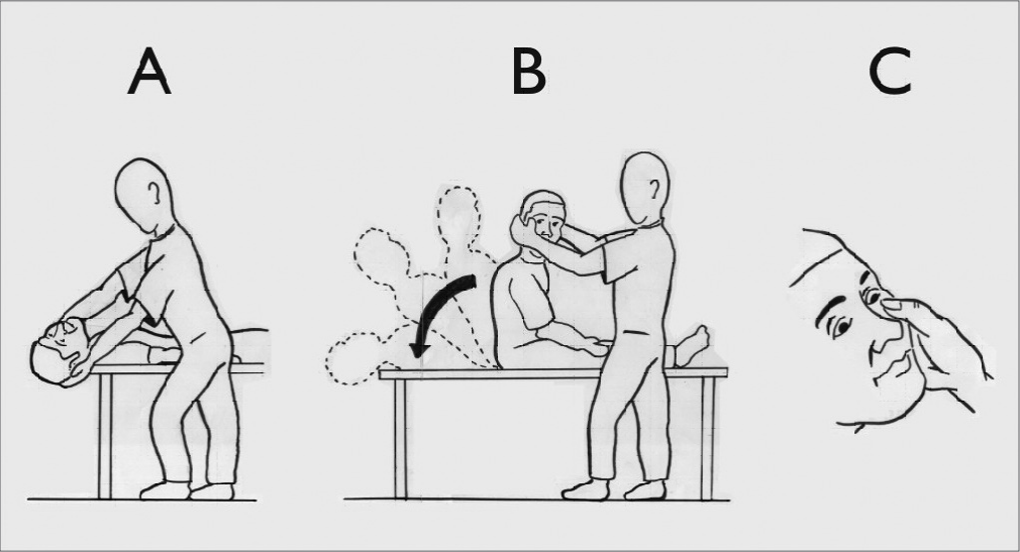
Physiopathological substrate and semicircular canal involved, according to the positional nystagmus characteristics at the Dix-Hallpike test (1952)^36^, in patients with BPPV, according to Ganança et al. (2000)^16^.

Patients were classified according to the posterior semicircular canal involved, indicated by the nystagmus triggering position and its direction. Chart 1 shows the canal involved according to the positional nystagmus characteristics presented at the Dix-Hallpike test^16^.

Treatment involved maneuvers to reposition statocones according to the semicircular canal involved. Epley’s maneuver was used, without the mastoid vibrator or patient sedation. The first maneuver was carried out immediately after neurotologic assessment.

Patients were subdivided in two groups. Group I was made up of patients who underwent one single Epley’s maneuver per weekly session, until positional nystagmus cessation at the Dix-Hallpike maneuver. Group II was made up of patients who underwent four Epley’s maneuvers in the first session, at two minute intervals, to one maneuver per week until positional nystagmus cessation at the Dix-Hallpike test. Both groups were compared in terms of clinical development. We considered the number of statocones re sessions necessary to eliminate positional nystagmus at the Dix-Hallpike test.

The data underwent statistical analysis. Qualitative analysis were expressed as as number and percentage and the quantitative ones as mean ± standard deviation for age, and as median (minimum and maximum) for months since symptoms onset, latency and nystagmus duration.

Chi-squared test was used to assess the association between qualitative variables, and, in those cases in which one of the frequencies expected were bellow five, we used the Fisher’s Exact Test, or its generalization. The student t test was used to compare the mean values and, in those cases in which data normality assumption was not met, we used the Mann-Whitney test. 5% was the significance value used, in other words, results with a p-value bellow 5% (p<0.05) were considered significant. Statistically significant results were marked with an asterisk.

## RESULTS

The study sample was made up of 123 patients, being 75 (61.0%) in group I and 48 (39.0%) in group II.

According to Table 1, groups I and II were homogeneous as for age and gender (p>0.05). In Group II, there was a higher proportion of patients classified as Caucasians when compared to Group I (p=0.012).

**Table 1:** Demographic data of the groups

		GROUPS	
Demographic data	I (n=75)	II (n=48)	p-value
Age	59,5 ± 15,6	55,3 ± 16,7	0,157+
Gender			0,385++
Male	21 (28,0%)	17 (35,4%)	
Female	54 (72,0%)	31 (64,6%)	
Race			0,012*+++
Caucasian	54 (72,0%)	44 (91,7%)	
Black	2 (2,7%)	1 (2,1%)	
Mixed	19 (25,3%)	3 (6,2%)	

Legend

+ t Student test

++ Chi-squared

+++ Generalization of the Exact Fisher’s Test

* Statistically significant

According to Table 2, Groups I and II were homogeneous as to the type of dizziness and the time of symptoms onset (p>0.05).

**Table 2:** Dizziness characteristics of the groups

		GROUPS	
Dizziness	I (n=75)	II (n=48)	p-value
Time of onset			0,166+
0 a 3 months	19 (25,3%)	22 (45,8%)	
4 a 6 months	12 (16,0%)	6 (12,5%)	
7 a 12 months	5 (6,7%)	2 (4,2%)	
1 a 4 years	20 (26,7%)	12 (25,0%)	
≥ 5 years	19 (25,3%)	6 (12,5%)	
Types			0,620+
Rotational	44 (58,7%)	25 (52,1%)	
Non-rotational	7 (9,3%)	7 (14,6%)	
Both	24 (32,0%)	16 (33,3%)	

Legend

+ Generalization of the Exact Fisher’s Test

According to Table 3, groups I and II were homogeneous as to the labyrinth involved and seen at the Dix-Hallpike test (p=0.276). Group II presented nystagmus latency and duration which were statistically higher when compared to Group I (p<0.05).

**Table 3:** Nystagmus characteristics in the groups.

		GROUPS	
Nystagmus	I (n=75)	II (n=48)	p-value
Labyrinth involved			0,276+
Right	41 (54,7%)	31 (64,6%)	
Left	34 (45,3%)	17 (35,4%)	
Latency	3,0 (1 - 20)	5,0 (1 - 30)	<0,001*++
Duration	6,0 (2 - 50)	10,0 (2 - 40)	0,005*++

Legend

+ Chi-squared test

++ Mann-Whitney test

* Statistically significant

One female patient from Group II had nausea and vomit right at the first session and was taken off the sample. In order to compare the number of sessions carried out, the groups were made up of 75 (61.5%) patients in group I and 47 (38.5%) in Group II.

Table 4 presents the descriptive measures of the number of sessions for each group. The mean value and the standard deviation for group I were higher when compared to those from group II. The Mann-Whitney test assessment found statistically significant differences as to the distribution of the number of sessions carried out in the two groups (p=0.008).

**Table 4:** Descriptive measures of the number of sessions per group.

		GROUPS	
Descriptive measures	I (n=75)	II (n=47)	p-value
Mean value	1,5	1,2	0,008 *+
Standard deviation	0,9	0,5	
Median	1,0	1,0	
Minimum	1	1	
Maximum	5	3	

Legend

+ Mann-Whitney test

* Statistically significant

Table 5 shows the distribution of the 122 patients according to the number of sessions necessary to abolish positional nystagmus at the Dix-Hallpike test. By means of the Fisher’s Exact Test we observed a statistically significant association between the distribution of the number of sessions and the group (p=0.039). Group II presented 21.4% more patients who needed only one session (CI 95% [7.7% - 35.1%]).

**Table 5:** Patients’ distribution according to the number of sessions and groups.

		GROUPS	
# of sessions	I (n=75)	II (n=47)	p-value
1	51 (68,0%)	42 (89,4%)	0,039 *+
2	17 (22,6%)	3 (6,4%)	
3	3 (4,0%)	2 (4,3%)	
4	2 (2,7%)	-	
5	2 (2,7%)	-	

Legend

+ Generalization of the Fisher’s Exact Test

* Statistically significant

The sample power with 75 patients in group I and 47 patients in Group II was of 80.0% in order to detect a difference of 0.214 between the null hypothesis, in which the proportion of patients who needed only one session in both groups was of 0.680 and the alternative hypothesis, in which the proportion in Group II was of 0.894, using the chi-squared test with a significance level of 5%.

**Figure 1:**
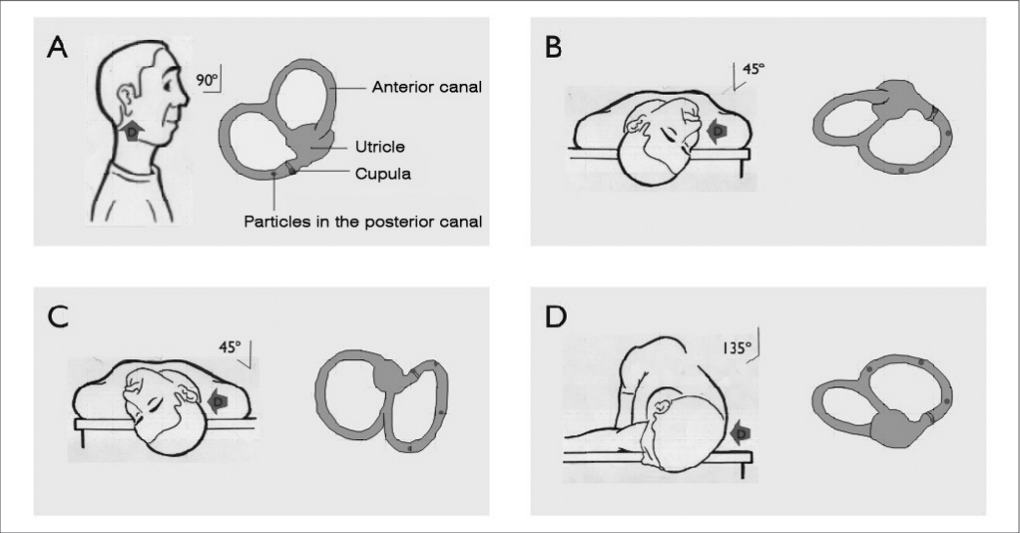
Dix-Hallpike test (1952)36: A) patient seating in bed, turns head 45º to the side being studied; B) with the help of the examiner, the patient quickly lays down on her back and keeps her head hanging, keeping head position at 45º to the side, and C) the examiner watches the patients eyes, looking for positional nystagmus.

**Figure 2:**
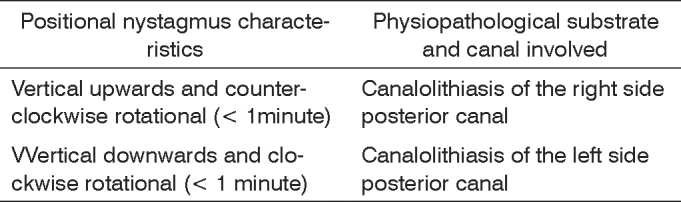
Statocones repositioning maneuver (right ear). A) patient seating in bed and with his head turned 45º to the right side; B) patient, with the examiner’s help, quickly lays down on his back and hangs his head, keeping the head turned. Statocone debris are shifted by gravity action in and away from the ampulla, causing canal deflection and nystagmus. This position is kept for one to two minutes. C) the patient’s head is then turned 90 degrees towards the non-involved side; D) the patient’s head and body are turned 90 degrees more towards the non-involved side. This position is kept for one to two minutes and the patient returns to his seating position. Statocone debris, by gravity, move to the utricle.

## DISCUSSION

In this study, group I, made up of patients who underwent one single Epley’s maneuver per weekly session until total remission of positional nystagmus and group II, made up of patients who underwent four Epley’s maneuvers in the first session and one weekly maneuver until positional nystagmus remission, they were homogeneous as far as age, gender, dizziness type and dizziness onset are concerned and as to the labyrinth involved at the Dix-Hallpike test. There was a higher proportion of Caucasian patients in group II, treated with repeated maneuvers per session.

Nystagmus latency and duration in Group II patients who underwent four maneuvers in the first session were higher than those in Group I, treated with one single maneuver in the first session, which could suggest a greater difficulty in resolution. Even with this apparent obstacle, patients from group II needed less sessions (1.2 sessions in average) of rehabilitation than the other group (1.5 session, in average), and the difference was statistically significant. The percentage of patients who needed only one session in order to get rid of positional nystagmus was 21.4% higher in Group II. Maneuver repetition in one single session was considered more convenient, since it allowed us to show the patient the treatment’s efficacy, confirmed by the progressive improvement in symptoms during the procedure, although we did not find significant differences between one single session with up to four maneuvers and one session with only one maneuver^35^. Since the patients did not undergo the same number of maneuvers in the first session^35^, it is possible to question if less than or more than the four maneuvers used in this study could have influenced the number of sessions necessary to stop positional nystagmus. A higher number of maneuvers could facilitate a more complete cleaning of debris in the semicircular canal involved.

Procedure tolerance was considered good in both groups we treated. Only one patient had nausea and vomits at maneuver repetition in one single session. The session with repeated maneuvers was well tolerated by the patients^35^.

Among the 123 patients in the present investigation, we did not see any case of posterior semicircular canal BPPV moving on to the anterior or horizontal canals after the maneuvers, which is in agreement with the findings of a study that used the single or repeated maneuvers^35^ and disagreeing from another study^37^, which found such conversion in 6.0% of the cases treated with one single maneuver.

In the literature studied we did not find other studies which compared the single maneuver versus the repeated maneuver in the first session, as to BPPV treatment efficacy and tolerance.

## CONCLUSION

Having the results we achieved, we can state that BPPV’s treatment by repeating Epley’s maneuver in one single session proved to be more efficient than one single maneuver per session.
